# Detection of Serum Levels of Interleukins-17, 21, 6 and Tumor Necrosis Factor-α in Patients with Patchy Alopecia Areata and Their Association with Disease Severity

**DOI:** 10.3390/diseases13090283

**Published:** 2025-09-01

**Authors:** Abeer Khaled Abu-El-Azayem, Zinab Alatawi, Helal F. Hetta, Ayman Salama, Nizar Sirag, Eid Alatwi, Rehab Mohamed Naguib, Randa Erfan, Haitham Abdelhamid, Abeer El-Dessouki El Sayed, Mera Mohamed Galal Anan

**Affiliations:** 1 Medical Microbiology and Immunology Department, Faculty of Medicine, Cairo University, Cairo 11956, Egypt; abeer.aboualazaim@kasralainy.edu.eg (A.K.A.-E.-A.); mira.hassan36203@postgrad.kasralainy.edu.eg (M.M.G.A.); 2 Department of Family and Community Medicine, Faculty of Medicine, University of Tabuk, Tabuk 47512, Saudi Arabia; zalatawi@ut.edu.sa; 3 Division of Microbiology, Immunology and Biotechnology, Department of Natural Products and Alternative Medicine, Faculty of Pharmacy, University of Tabuk, Tabuk 71491, Saudi Arabia; 4 Department of Pharmaceutics, Faculty of Pharmacy, University of Tabuk, Tabuk 71491, Saudi Arabia; agrawan@ut.edu.sa; 5 Division of Pharmacognosy, Department of Natural Products and Alternative Medicine, Faculty of Pharmacy, University of Tabuk, Tabuk 71491, Saudi Arabia; nmona@ut.edu.sa; 6 Department of Pharmacology, College of Pharmacy, Jouf University, Sakaka 72388, Saudi Arabia; esatawi@ju.edu.sa; 7 Dermatology and Venerology Department, Faculty of Medicine, Beni-Suef University, Beni-Suef 62511, Egypt; rehab.mohamed@med.bsu.edu.eg; 8 Department of Medical Biochemistry and Molecular Biology, Faculty of Medicine, Cairo University, Cairo 11956, Egypt; randa.erfan@cu.edu.eg; 9 Plastic Surgery and Hair Transplantation Center, Vertex Ästhetik Klinik, Cairo 12566, Egypt; mylife4eve@gmail.com; 10 Health Administration and Information Technology, Faculty of Applied Health Science Technology, Misr University for Science and Technology, 6th of October City, Giza 12566, Egypt; abeer.eldessoki@must.edu.eg

**Keywords:** alopecia areata, cytokines, interleukins, inflammation

## Abstract

**Background/Objectives**: Alopecia areata (AA) is a common autoimmune disorder characterized by non-scarring hair loss. The destruction of hair follicle cells is mediated by cytotoxic T cells, which release cytokines that contribute to tissue damage. Among these, Th17 cells produce key inflammatory mediators, including IL-17, IL-21, IL-6, and TNF-α, which may play a role in disease progression. This study aimed to evaluate the serum levels of IL-17A, IL-21, IL-6, and TNF-α in patients with patchy AA compared with healthy controls and to assess their association with disease severity. **Methods**: A cross-sectional analytical study was conducted on 50 patients with patchy AA and 50 age- and gender-matched healthy controls. Blood samples were collected, and serum cytokine levels were measured using enzyme-linked immunosorbent assay (ELISA) kits. **Results**: Of the patients, 70% were female (35 patients) and 30% were male (15 patients). Disease severity distribution showed that 66% had mild AA, 22% had moderate AA, and 12% had severe AA. Compared with controls, AA patients exhibited significantly elevated serum levels of IL-6, TNF-α, IL-17A, and IL-21 (*p* < 0.001). Additionally, cytokine levels showed a positive correlation with both age and disease duration, suggesting their potential role in disease progression. **Conclusions**: These findings highlight the involvement of pro-inflammatory cytokines in the pathogenesis of patchy AA and their correlation with disease severity. Understanding these cytokine interactions may provide insights into targeted therapeutic strategies in managing AA.

## 1. Introduction

Alopecia areata (AA) is an organ-specific autoimmune disease characterized by non-scarring hair loss due to a T-cell-mediated attack on hair follicle (HF) autoantigens [1]. The hair follicle is typically an immune-privileged site with low major histocompatibility complex (MHC) expression. However, AA arises when this immune privilege is disrupted, leading to an autoimmune assault on the follicular bulb by CD8+ T lymphocytes. The resulting peribulbar lymphocytic infiltrate triggers apoptosis in hair follicle keratinocytes, halting cell division within the hair matrix and suppressing hair shaft production [2].

AA manifests in three main clinical subtypes: patchy AA, alopecia totalis (AT, complete scalp hair loss), and alopecia universalis (AU, complete loss of body hair) [3]. Disease severity is commonly assessed using the Severity of Alopecia Tool (SALT), which categorizes hair loss extent into standardized quadrants (S0–S5) [4]. S0 indicates no hair loss, S1 indicates 1–24% hair loss, S2 indicates 25–49% hair loss, S3 indicates 50–74% hair loss, S4 indicates 75–99% hair loss, and S5 indicates 100% hair loss.

The immune-mediated destruction of hair follicle cells may occur through direct cytotoxic mechanisms, such as the Fas and perforin pathways, or indirectly via the release of pro-inflammatory cytokines [5]. Among these, Th17 cells—defined by their ability to produce IL-17, IL-21, IL-6, and TNF-α—play a key role in AA pathogenesis [6]. The IL-17 pathway has been implicated in various inflammatory and autoimmune conditions, including psoriasis, rheumatoid arthritis, multiple sclerosis, and systemic sclerosis. While prior studies have reported elevated serum levels of IL-17, IL-21, IL-6, and TNF-α in AA patients, their specific association with disease severity remains insufficiently explored [7].

Despite growing evidence implicating cytokines in the pathogenesis of AA, the specific relationship between key pro-inflammatory mediators and disease severity remains insufficiently explored. While prior studies have reported elevated levels of these cytokines in AA patients, their correlation with clinical severity and disease progression is not well established. Understanding these associations is crucial for identifying potential biomarkers for disease monitoring and targeted therapeutic strategies [8,9]. Therefore, this study aims to evaluate the serum levels of IL-17, IL-21, IL-6, and TNF-α in patients with patchy AA compared with healthy controls and to investigate their correlation with disease severity, providing insights into the immunopathogenesis of AA.

## 2. Patients and Methods

### 2.1. Ethical Statement

Informed consent was obtained from patients. The study was conducted under the tenets of the Declaration of Helsinki and with approval from the Ethical Committee of the Faculty of Medicine, Cairo University, Egypt (IRB No.N-101-2024).

### 2.2. Study Design and Population

This cross-sectional analytical study was conducted on 50 patients diagnosed with patchy alopecia areata (AA) and 50 age- and gender-matched healthy controls with no clinical evidence or history of dermatological, autoimmune, or chronic inflammatory diseases. Prior to inclusion, all control participants completed a structured medical questionnaire and underwent a brief physical examination to exclude any underlying systemic or immune-related conditions. Individuals reporting recent infections, ongoing medication use, or a family history of autoimmune disorders were also excluded to ensure a homogenous, immunologically healthy control group. Patients were recruited from the Dermatology and Venereology Department, Faculty of Medicine, Beni-Suef University, between February 2024 and August 2024.

### 2.3. Inclusion and Exclusion Criteria

Inclusion criteria: Patients diagnosed with patchy AA who had not received systemic or topical treatment for at least one month prior to enrollment. Both male and female patients were included.

Exclusion criteria: Individuals with other dermatological, autoimmune, or systemic diseases were excluded to minimize confounding variables.

### 2.4. Sample Collection and Laboratory Analysis

A 3 mL peripheral venous blood sample was collected from each participant. Serum was separated by centrifugation and stored at −70 °C until further analysis. Serum concentrations of IL-17A, IL-21, IL-6, and TNF-α were measured using enzyme-linked immunosorbent assay (ELISA) kits according to the manufacturers’ protocols. IL-17A levels were assessed using a kit from STEMCELL Technologies (Vancouver, BC, Canada), IL-21 from Cusabio Biotech, Life Sciences Advanced Technologies Inc., St. Petersburg, FL, USA, and IL-6 and TNF-α from Elabscience Biotechnology, Houston, TX, USA. All laboratory work was conducted at the Microbiology and Immunology Department, Kasr Al Ainy, Cairo University.

### 2.5. Statistical Analysis

Data analysis was performed using SPSS software version 28 (IBM Corp., Armonk, NY, USA). Continuous variables were summarized as mean ± standard deviation (SD), median, and range, while categorical variables were presented as frequencies and percentages. Comparisons between groups were carried out using unpaired *t*-tests for normally distributed data and analysis of variance (ANOVA) with post hoc tests for multiple group comparisons. For non-normally distributed data, the Kruskal–Wallis and Mann–Whitney U tests were applied. Categorical variables were compared using the Chi-square test or Fisher’s exact test when applicable. Correlations between cytokine levels, disease severity, and clinical parameters were analyzed using Spearman’s correlation coefficient. A *p* value of less than 0.05 was considered statistically significant.

### 2.6. Sample Size and Power Analysis

This study was exploratory in nature; therefore, a priori sample size calculation was not performed. However, a post hoc power analysis was conducted using the observed mean differences and standard deviations for the primary cytokines (IL-6, TNF-α, IL-17A, and IL-21) between the AA and control groups. The calculated power exceeded 80% for each cytokine, indicating that the sample size was adequate to detect statistically significant differences at α = 0.05.

## 3. Results

### 3.1. Demographic and Clinical Characteristics of Alopecia Areata Patients

This study included 50 patients diagnosed with patchy alopecia areata (AA) and 50 age- and gender-matched healthy controls. Among the AA patients, 70% (35 patients) were female, and 30% (15 patients) were male. The age of the patients ranged from 6 to 50 years, with a mean of 24.14 ± 11.03 years.

In addition to age, sex, and disease duration, relevant clinical and lifestyle variables were assessed. A positive family history of alopecia areata was reported in 10% of the cases. Regarding lifestyle factors, 7% of the patients were current smokers, while none reported alcohol consumption. Sleep disturbances, including insomnia and poor sleep quality, were reported by 15% of the participants. These factors may play a contributory role in disease susceptibility or severity and may require further investigations in future research.

Regarding disease severity, 66% (33 patients) had mild AA, 22% (11 patients) had moderate AA, and 12% (6 patients) had severe AA. Facial hair loss was present in 24% (12 patients), while 76% (38 patients) did not exhibit facial involvement. Additionally, 22% (11 patients) reported a history of three or more disease relapses, indicating a recurrent disease pattern in a subset of patients. These demographic and clinical characteristics are summarized in Table 1.

### 3.2. Comparison of Cytokine Levels Between Patients and Controls

**Figure 1:**
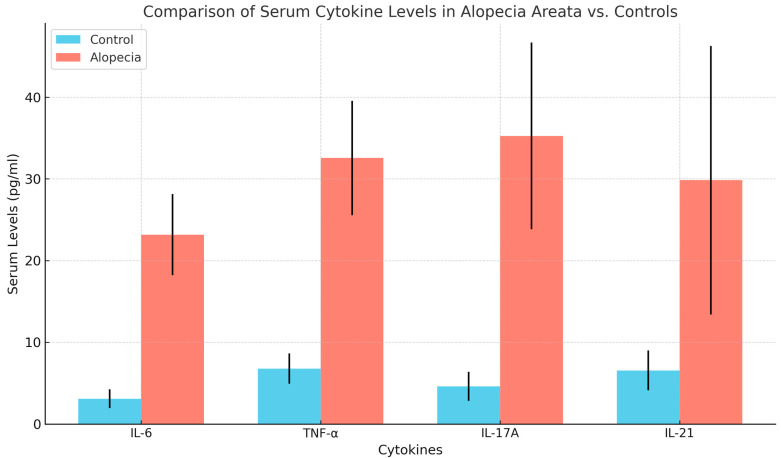
Comparison of serum cytokine levels between cases and control.

Serum levels of inflammatory cytokines were significantly higher in AA patients compared with the control group. Serum levels of IL-6 (23.16 ± 4.97 pg/mL), TNF-α (32.56 ± 7.00 pg/mL), IL-17A (35.26 ± 11.44 pg/mL), and IL-21 (29.85 ± 16.45 pg/mL) were significantly elevated in AA patients compared with healthy controls (IL-6: 3.11 ± 1.16 pg/mL, TNF-α: 6.78 ± 1.84 pg/mL, IL-17A: 4.63 ± 1.78 pg/mL, IL-21: 6.56 ± 2.44 pg/mL), with all differences being statistically significant (*p* < 0.001). These findings are detailed in Table 2 and visually represented in Figure 1.

### 3.3. Correlation of Cytokine Levels with Age and Disease Duration

**Figure 2:**
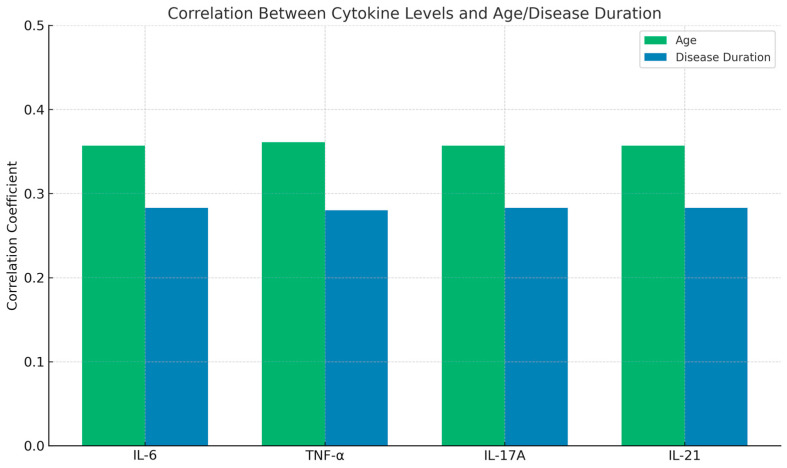
Correlation of cytokines with age and disease duration.

Further analysis revealed a positive correlation between cytokine levels and both age and disease duration. Higher serum levels of IL-6, TNF-α, IL-17A, and IL-21 were observed in older patients and those with a longer disease duration. These correlations were statistically significant (*p* < 0.05), as shown in Table 3 and Figure 2. These findings suggest that inflammatory cytokines may play a role in disease progression and severity over time.

### 3.4. Association Between Cytokine Levels and Disease Severity

**Figure 3:**
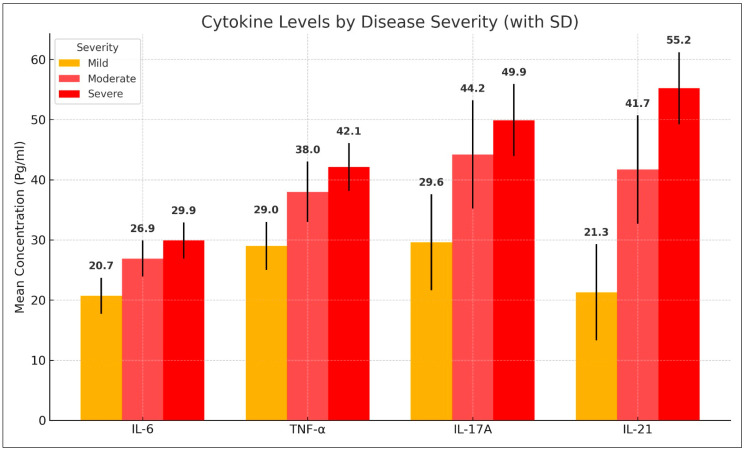
Comparison of cytokine levels among alopecia areata patients with different disease severity.

Cytokine levels varied significantly across different severity groups. Patients with mild AA had lower levels of IL-6, TNF-α, IL-17A, and IL-21 compared with those with moderate and severe AA. The mean serum levels of IL-6 increased from 20.66 ± 3.36 pg/mL in mild cases to 26.93 ± 4.36 pg/mL in moderate cases and 29.95 ± 2.11 pg/mL in severe cases. Similarly, TNF-α levels rose from 29.00 ± 4.93 pg/mL in mild cases to 38.03 ± 5.56 pg/mL in moderate cases and 42.07 ± 2.26 pg/mL in severe cases. IL-17A and IL-21 followed a similar trend, with significantly elevated levels in more severe cases (*p* < 0.001). These findings are summarized in Table 4 and Figure 3.

Post hoc pairwise comparisons further confirmed these differences, revealing statistically significant elevations in cytokine levels between mild and moderate cases, as well as between mild and severe cases (*p* < 0.001). However, no statistically significant differences were observed between moderate and severe cases (*p* > 0.05), suggesting a plateau in cytokine elevation at higher disease severity levels. These comparative analyses are detailed in Table 5.

### 3.5. Correlation Between Cytokine Levels

A strong positive correlation was observed among all measured cytokines, indicating a tightly interconnected inflammatory response. IL-6 and TNF-α exhibited a near-perfect correlation (r = 0.994, *p* < 0.001), while IL-6 and IL-17A showed an almost identical relationship (r = 1.000, *p* < 0.001). Similarly, IL-17A and TNF-α were highly correlated (r = 0.994, *p* < 0.001), and IL-21 demonstrated a perfect correlation with both IL-17A (r = 1.000, *p* < 0.001) and IL-6 (r = 1.000, *p* < 0.001), as outlined in Table 6.

A strong positive correlation (r > 0.9) indicates that as the levels of one cytokine increase, the levels of the others rise in a nearly proportional manner, reinforcing the notion of a coordinated inflammatory response in AA pathogenesis.

No statistically significant differences were observed in cytokine levels between male and female patients. The mean serum levels of IL-6, TNF-α, IL-17A, and IL-21 were comparable between the two sexes, with *p* values exceeding 0.05, indicating that gender did not appear to influence the systemic inflammatory response in alopecia areata. These findings are summarized in Table 7.

## 4. Discussion

Alopecia areata (AA) is a complex autoimmune condition characterized by patchy hair loss, with an underlying pathogenesis driven by immune dysregulation [10]. This study demonstrated significantly elevated levels of IL-6, TNF-α, IL-17A, and IL-21 in AA patients compared with healthy controls, reinforcing the role of pro-inflammatory cytokines in disease development and progression. The strong correlation between cytokine levels and disease severity further supports their involvement in the immunopathological mechanisms underlying AA.

The increased serum levels of IL-6, TNF-α, IL-17A, and IL-21 in patch-type alopecia areata suggest a coordinated pro-inflammatory immune response. These cytokines are known to be involved in key intracellular signaling pathways that disrupt hair follicle immune privilege and promote autoimmune-mediated damage [10].

IL-6 acts through the JAK/STAT3 pathway, promoting the differentiation of Th17 cells, which are central in autoimmune responses. Elevated IL-17A, produced by these Th17 cells, further amplifies local inflammation by inducing keratinocytes and dermal fibroblasts to secrete additional chemokines and cytokines, perpetuating T-cell recruitment to the hair follicle [10].

TNF-α, a major effector cytokine, activates NF-κB signaling, leading to increased expressions of adhesion molecules and inflammatory mediators that facilitate T-cell infiltration and follicular destruction. IL-21, also produced by Th17 and Th cells, enhances the proliferation of autoreactive T cells and sustains chronic inflammation by reinforcing IL-17 expression through STAT3 activation [10].

These mechanisms likely act synergistically to compromise the immune privilege of anagen hair follicles, rendering them susceptible to cytotoxic T-cell attack. Understanding these intracellular pathways underscores the potential of targeted immunomodulatory therapies (e.g., JAK inhibitors) in altering the disease course of patchy AA [10].

AA may affect both women and men; in our study 70% of the patients were female (35 patients) and 30% were male (15 patients); this result aligns with most studies in which AA has been reported to be more common in females [11]; however, there are few studies in which AA was reported to be more common in males [12]. This could be explained by the general female predominance in autoimmune disorders.

AA was reported to be more common in younger age groups than adults [13]. Our study showed that the average age of participants in the control group was significantly higher (29.66 ± 10.79 years) than the alopecia group (24.14 ± 11.03 years). Our findings are consistent with those of Seyrafi et al., who documented a median age of 24.05 ± 9.98 years in patients with AA, and Kavak et al., who reported a comparable value of 24.32 ± 0.54 years [14,15]. In contrast, Uzuncakmak et al. observed higher median ages: 29.86 ± 14.48 years for AA, 29.50 ± 16.18 years for AT, and 32.81 ± 14.48 years for AU. They attributed the increased median age in AA to the tendency of the condition to progress to AU over time [11].

Consistent with previous studies, our findings align with reports indicating increased serum levels of IL-6, IL-17, IL-21, and TNF-α in AA patients, highlighting the role of Th17-related cytokines in disease activity. Atwa et al. reported that serum concentrations of IL-6, IL-17, IL-21, IL-22, and TNF-α were markedly elevated in AA patients [16]. In a comparable finding, Bain et al. observed higher levels of IL-1β, IL-6, and TNF-α in this patient group [17]. Likewise, Tabara et al. found that IL-6, IL-15, IL-17A, and IFN-γ were significantly increased in the serum of individuals with AA compared with healthy controls [18]. However, Morsy et al. detected no difference in the serum level of IL-17 between patients with alopecia areata and healthy controls [19].

These results indicate that subjects with alopecia have a lower average age, and significantly higher levels of pro-inflammatory cytokines compared with the control group. This could be explained by the pathophysiology of AA, which is influenced by the high levels of these cytokines, which are essential in the inflammatory processes linked to autoimmune disorders.

Our study also revealed a positive correlation between the levels of the detected cytokines and disease duration (*p* < 0.05).

Previous studies have indicated that the serum concentrations of several cytokines, such as IL-6 and TNF-α, may be linked to disease duration. Tomaszewska et al. reported a positive association between IL-6 levels and the length of alopecia areata [20]. Similarly, Rossi et al. found that TNF-α levels correlated positively with the duration of the disease [21] but Kasumagić-Halilović reported that no correlations were found between the duration of disease and the serum levels of TNF-α (*p* = 0.7361) [22]. Moreover, the serum level of IL-17 decreased in patients with the current episode of hair loss longer than 2 years [18].

The significant elevation in cytokines in patients with AA, particularly in those with more severe forms of the disease, suggests a correlation between the severity of hair loss and the inflammatory response. Our results indicated that as the severity of alopecia increased from mild to severe, there was a corresponding increase in the levels of IL-6, TNF-α, IL-17A, and IL-21. This aligns with previous studies that have reported heightened levels of these cytokines in various inflammatory conditions and autoimmune diseases. Studies have shown a positive relationship between alopecia areata severity and serum concentrations of several cytokines, including IL-2, TNF, IL-12, IL-17, and IL-17E [8]. These findings suggest that the intensity of systemic inflammation may influence not only severity but also the persistence of hair loss in affected individuals. Consequently, patients presenting with more extensive forms—such as AT or AU—or with prolonged disease duration may be more susceptible to the systemic complications associated with chronic inflammation.

Although 70% of the study participants were female, statistical comparisons of serum cytokine levels (IL-6, TNF-α, IL-17A, and IL-21) between male and female patients revealed no significant differences (*p* > 0.05 for all). This suggests that the elevated prevalence of patch-type alopecia areata in women may be influenced by factors other than the systemic inflammatory cytokines examined in this study [21]. Therefore, while cytokine dysregulation plays a role in alopecia areata pathogenesis, it does not appear to account for sex-specific differences in disease presentation.

The marked elevation in IL-6, TNF-α, IL-17A, and IL-21 in patients with patch-type alopecia areata not only underscores their role in disease pathogenesis but also supports their potential as clinically relevant biomarkers. The positive correlations between these cytokines and both patient age and disease duration suggest a possible link with disease activity or chronicity. This raises the possibility of using these markers for monitoring disease progression or evaluating therapeutic response over time [23].

Emerging evidence suggests that elevated IL-6 and IL-17A may indicate a Th17-skewed inflammatory environment, which is responsive to immunomodulatory agents such as JAK inhibitors or IL-17/IL-6 pathway blockers. Moreover, TNF-α and IL-21 have also been implicated in autoimmune-driven tissue damage and could represent targets for monoclonal antibody therapies [23].

Thus, profiling cytokine levels in AA patients could guide personalized therapeutic strategies, particularly in selecting patients who may benefit from targeted biologics or in assessing response to treatment. Further longitudinal studies are warranted to validate these cytokines as reliable indicators of disease activity and treatment efficacy [23].

### Recommendation

Future research should focus on longitudinal studies to better understand the temporal relationship between cytokine elevation and disease progression in alopecia areata. Investigating cytokine fluctuations over time could help establish their role as predictive biomarkers for disease activity and treatment response.

Additionally, targeted immunotherapies should be explored, particularly biologic agents that inhibit IL-6, TNF-α, IL-17A, or IL-21 pathways. Given the success of cytokine inhibitors in other autoimmune diseases, clinical trials evaluating their efficacy and safety in AA patients are warranted.

Further studies should also examine genetic and molecular mechanisms underlying cytokine dysregulation in AA, particularly the interplay between Th17-driven inflammation and hair follicle immune privilege collapse. Identifying key regulatory pathways could lead to more precise therapeutic interventions.

Lastly, personalized treatment strategies integrating cytokine profiling with clinical severity assessments may improve disease management. Developing a standardized cytokine-based scoring system could aid in stratifying patients and tailoring immunomodulatory therapies accordingly.

## 5. Conclusions

Our study highlights the significant elevation in IL-6, TNF-α, IL-17A, and IL-21 in AA patients and their strong correlation with disease severity and duration. The robust interplay among these cytokines underscores their role in AA pathogenesis and suggests their potential as biomarkers for disease monitoring. These findings pave the way for further investigations into cytokine-targeted therapies, which may offer new avenues for improving patient outcomes.

## Figures and Tables

**Table 1:** Demographic and clinical characteristics of alopecia areata patients.

		ALOPECIA	
		Count	%
Sex	Female	35	70.0%
	Male	15	30.0%
Disease severity	Mild	33	66.0%
	Moderate	11	22.0%
	Severe	6	12.0%
Relapses of 3 or more	Yes	11	22.0%
	No	39	78.0%
Facial hair	Yes	12	24.0%
	No	38	76.0%

**Table 2:** Cytokine level comparison between cases and control.

	Control					ALOPECIA					*p* Value
	Mean	SD	Median	Minimum	Maximum	Mean	SD	Median	Minimum	Maximum	
IL-6 (pg/mL)	3.11	1.16	2.33	1.49	5.01	23.16	4.97	22.81	15.31	33.09	<0.001
TNF-α (pg/mL)	6.78	1.84	5.93	4.12	10.13	32.56	7.00	31.98	20.55	45.01	<0.001
IL-17A (pg/mL)	4.63	1.78	4.11	1.79	8.91	35.26	11.44	34.87	17.54	56.85	<0.001
IL-21 (pg/mL)	6.56	2.44	4.92	3.14	10.56	29.85	16.45	21.64	13.79	62.21	<0.001

**Table 3:** Correlation of cytokines with age and disease duration.

		IL-6 (pg/mL)	TNF-α (pg/mL)	IL-17A (pg/mL)	IL-21 (pg/mL)
Age	Correlation coefficient	0.357	0.361	0.357	0.357
	*p* value	0.011	0.010	0.011	0.011
	N	50	50	50	50
Disease duration in months	Correlation coefficient	0.283	0.280	0.283	0.283
	*p* value	0.046	0.049	0.046	0.046
	N	50	50	50	50

**Table 4:** Comparison of cytokine levels among alopecia areata patients with different disease severity classifications.

	Disease Severity															
	Mild					Moderate					Severe					*p* Value
	Mean	SD	Median	Minimum	Maximum	Mean	SD	Median	Minimum	Maximum	Mean	SD	Median	Minimum	Maximum	
IL-6 (pg/mL)	20.66	3.36	19.74	15.31	29.04	26.93	4.36	27.16	20.73	33.09	29.95	2.11	29.91	27.16	33.09	<0.001
TNF-α (pg/mL)	29.00	4.93	27.63	20.55	41.64	38.03	5.56	39.04	30.21	45.01	42.07	2.26	42.43	39.04	45.01	<0.001
IL-17A (pg/mL)	29.62	8.53	29.39	17.54	48.33	44.18	8.74	44.90	31.93	56.85	49.94	4.48	49.40	44.90	56.85	<0.001
IL-21 (pg/mL)	21.30	8.20	18.09	13.79	54.58	41.67	17.61	44.38	18.66	62.21	55.20	6.27	56.22	44.38	62.21	<0.001

**Table 5:** Post hoc pairwise comparisons of cytokine levels between different disease severity groups in alopecia areata patients.

	Mild Versus Moderate	Mild Versus Severe	Moderate Versus Severe
IL6 (pg/mL)	<0.001	<0.001	0.288
TNF-α (pg/mL)	<0.001	<0.001	0.324
IL-17A (pg/mL)	<0.001	<0.001	0.525
IL-21 (pg/mL)	0.001	<0.001	0.998

**Table 6:** Correlation of cytokines with each other.

		IL6 (pg/mL)	TNF-α (pg/mL)	IL-17A (pg/mL)
TNF-α (pg/mL)	Correlation Coefficient	0.994		
	*p* Value	<0.001		
	N	50		
IL-17A (pg/mL)	Correlation Coefficient	1.000	0.994	
	*p* Value	<0.001	<0.001	
	N	50	50	
IL-21 (pg/mL)	Correlation Coefficient	1.000	0.994	1.000
	*p* Value	<0.001	<0.001	<0.001
	N	50	50	50

**Table 7:** Comparison of serum cytokine levels between male and female alopecia areata patients.

	Sex										
	Male					Female					*p* Value
	Mean	SD	Median	Minimum	Maximum	Mean	SD	Median	Minimum	Maximum	
IL6 (pg/mL)	22.77	5.19	21.63	15.31	33.09	24.06	4.47	23.89	17.95	33.09	0.406
TNF-α (pg/mL)	31.99	7.39	29.85	20.55	45.01	33.87	6.03	32.95	25.13	45.01	0.389
IL-17A (pg/mL)	34.22	11.94	33.02	17.54	56.85	37.71	10.11	37.19	20.52	56.85	0.328
IL-21 (pg/mL)	29.23	16.86	19.47	13.79	62.21	31.29	15.92	27.43	15.99	62.21	0.324

## Data Availability

Data are contained within the article.
